# Comparative Assessment of Immune Cell Subset Ratios (NKT/NK, Th/Tc, B1/B2) in Gestational Diabetes and Healthy Pregnancy: Links to Biochemical and Immunochemical Profiles

**DOI:** 10.3390/metabo15060378

**Published:** 2025-06-08

**Authors:** Jelena Omazić, Andrijana Muller, Mirta Kadivnik, Blaženka Dobrošević, Barbara Vuković, Mirela Florijančić, Jasenka Wagner

**Affiliations:** 1 Department of Laboratory and Transfusion Medicine, “Dr. Juraj Njavro” National Memorial Hospital Vukovar, 32000 Vukovar, Croatia; jelena.omazic@gmail.com; 2 Department of Medical Chemistry, Biochemistry and Clinical Chemistry, Faculty of Medicine, J.J. Strossmayer University, 31000 Osijek, Croatia; 3 Clinic of Obstetrics and Gynecology, University Hospital Center Osijek, 31000 Osijek, Croatia; 4 Department of Obstetrics and Gynecology, Faculty of Medicine, J.J. Strossmayer University, 31000 Osijek, Croatia; 5 Institute of Clinical Laboratory Diagnostics, University Hospital Centre Osijek, 31000 Osijek, Croatia; 6 Poliklinika Retfala, 31000 Osijek, Croatia; 7 Department of Medical Biology and Genetics, Faculty of Medicine, J.J. Strossmayer University, 31000 Osijek, Croatia

**Keywords:** gestational diabetes, pregnancy, lymphocytes, immune regulation, flow cytometry, metabolic status

## Abstract

**Introduction:** Gestational diabetes (GD) is a common pregnancy metabolic disorder involving immune alterations. While there is a link between immune cells and GD, the specific roles of NKT/NK, helper/cytotoxic T, and B1/B2 lymphocyte ratios in complicated/uncomplicated pregnancies with and without GD are underexplored. This cross-sectional study hypothesized that specific imbalances in these lymphocyte ratios would be present in GD, and that these ratios would correlate with key metabolic parameters in pregnancy. **Methods:** We compared these lymphocyte ratios in 162 third-trimester pregnant women across four groups: healthy uncomplicated (*n* = 40), healthy complicated (*n* = 40), GD uncomplicated (*n* = 42), and GD complicated (*n* = 40), using flow cytometry and by measuring biochemical parameters. **Results:** No significant differences in lymphocyte ratios were found between GD and healthy pregnancies. Novel correlations emerged: in the entire cohort, the NKT/NK ratio positively correlated with C-peptide and triglycerides, and negatively with HDL cholesterol. The helper/cytotoxic ratio negatively correlated with insulin and C-peptide. In the GD group, NKT/NK correlated positively with C-peptide, and helper/cytotoxic negatively with insulin. **Conclusion:** These findings suggest a subtle yet significant link between immune cell subsets and metabolic status in pregnancy and GD, warranting further investigation.

## 1. Introduction

Gestational diabetes (GD) is the most prevalent metabolic disorder and medical complication encountered during pregnancy. It is defined by the initial recognition of hyperglycemia during gestation, encompassing both undiagnosed type 2 diabetes mellitus (T2DM) in early pregnancy and true GD that develops later [1,2]. GD is increasingly recognized as a condition characterized by alterations in the maternal immune system, with lymphocytes, macrophages, and eosinophils implicated in aberrant immune regulation [3].

B-lymphocytes (CD19+), a primary population of lymphocytes, play a significant role in both innate and adaptive immunity through antibody production and cytokine secretion. Prior research indicates that B-lymphocytes infiltrate visceral adipose tissue prior to T-lymphocytes, contributing to the regulation of insulin resistance alongside neutrophils, granulocytes, and macrophages. Murine studies involving high-fat diets have demonstrated elevated B-lymphocyte levels, suggesting an activation of the immune response [4]. The adaptive immune response, involving B-lymphocytes, is known to modulate the development of obesity and insulin resistance. Furthermore, B-lymphocytes influence T-lymphocyte activity and insulin sensitivity through the secretion of various cytokines, including interleukin 6 (IL-6), IL-10, and leptin [5]. Studies have also shown the direct impact of obesity and hyperglycemia on antibody production, which can modulate the degree of insulin resistance [6]. B-lymphocytes are broadly classified into two main subsets, B1 (CD19+CD5+) and B2 (CD19+CD5-) lymphocytes, distinguished by their origin, development, anatomical distribution, and T-lymphocyte dependence for antibody production [7,8]. Research suggests that the B1/B2-lymphocyte ratio is lower in the bone marrow, spleen, and subcutaneous adipose tissue compared to visceral adipose tissue, a site implicated in the pathogenesis of metabolic syndrome and insulin resistance. Notably, a reduced B1/B2 ratio has been observed in T2DM [9]. A healthy pregnancy typically presents with a reduced overall percentage of B-lymphocytes, but an increased proportion of regulatory B-lymphocytes, which exert protective and anti-inflammatory effects. Conversely, GD has been associated with an increased percentage of B-lymphocytes and a positive correlation with insulin resistance and elevated levels of immunoglobulin A (IgA), an antibody linked to adipose tissue inflammation and disrupted glucose homeostasis in obesity [10,11].

T-lymphocytes, the principal cellular components of the adaptive immune system, are responsible for cell-mediated immune responses. CD4+ helper T-lymphocytes (Th) represent a heterogeneous group central to virtually all aspects of the immune response. Upon activation, CD4+ T-lymphocytes can differentiate into various subsets, including Th1 and Th2 lymphocytes. A normal pregnancy is characterized by a prevalence of Th2 lymphocytes, crucial for maintaining maternal–fetal tolerance and associated with favorable pregnancy outcomes. However, conditions such as gestational diabetes, insulin resistance, or preeclampsia have been linked to a dominance of pro-inflammatory Th1 lymphocytes, potentially leading to adverse pregnancy outcomes [12,13].

Natural killer (NK) cells are inherently cytotoxic lymphocytes that, unlike cytotoxic T-lymphocytes, do not require prior antigen exposure to exert their antitumor effects [14,15]. Prior research has indicated impaired NK cell function upon exposure to hyperglycemia in diabetic patients, along with associations with reduced physical activity and poor metabolic status. Individuals with T1DM and T2DM often exhibit a lower percentage of NK cells compared to healthy populations [16]. NK cells are present in the decidua of the placenta and are essential for successful spiral artery remodeling and fetal implantation during the first trimester of pregnancy [17]. Studies have shown a decrease in the percentage of NK cells during the third trimester. An increased number of NK cells with a cytotoxic profile has been observed in pregnant women with GD compared to those without [13].

Natural killer T (NKT) cells are T-lymphocytes that share the characteristics of both NK cells and classical T cells, constituting approximately 0.2% of circulating T-lymphocytes. They recognize specific CD1d molecules presenting sugar lipids and polypeptides within histocompatibility complexes [18]. NKT cells are generally less abundant in peripheral blood during pregnancy. However, they accumulate in the decidual tissue, where their numbers are ten-fold higher than in the peripheral blood of pregnant women, and produce IFN-γ and IL-4, contributing to the protective environment at the fetal–maternal interface and maintaining the Th1/Th2 balance. Nevertheless, excessive NKT cell stimulation can potentially lead to spontaneous abortion. In the late stages of pregnancy, an increased activation of peripheral NKT cells secreting IL-4 has been noted. NKT cell subsets exhibit diverse effects on obesity-induced inflammation; they can promote insulin resistance by secreting inflammatory cytokines, particularly in response to excess lipids associated with obesity, but can also enhance insulin sensitivity through the secretion of Th2-type cytokines [13].

Given the complex interplay of these immune cell populations in the context of pregnancy and metabolic disorders, this research aimed to determine whether pregnant women with GD exhibit a higher ratio of NKT/NK cells and B1/B2 lymphocytes, and a lower ratio of helper/cytotoxic lymphocytes compared to healthy pregnant women. Furthermore, the study sought to investigate the relationship between these specific lymphocyte ratios and routine biochemical and immunochemical parameters. An imbalance in the NKT/NK cell ratio, for instance, could signal the dysregulation of immune tolerance, which is crucial for a healthy pregnancy, potentially contributing to the pro-inflammatory environment and insulin resistance seen in GD. Similarly, shifts in the B1/B2 lymphocyte ratio might indicate altered humoral immune responses, while an imbalanced helper/cytotoxic lymphocyte ratio could point to skewed T cell responses, both of which are implicated in chronic inflammation and metabolic dysfunction. By exploring these ratios and their correlation with routine biochemical and immunochemical parameters, our study aims to shed light on the underlying immunological mechanisms of GD, an area that has, to date, received insufficient research attention. This deeper understanding could ultimately pave the way for identifying predictive immune signatures and developing novel therapeutic strategies for GDM.

## 2. Materials and Methods

This cross-sectional study was conducted in accordance with the ethical principles outlined in the Nuremberg Code and the latest revision of the Declaration of Helsinki [19]. All participants provided written informed consent after reading a detailed description of the research. The study was conducted at the Clinic of Gynecology and Obstetrics and Department of Clinical Laboratory Diagnostic of the University Hospital Centre Osijek and Department for Laboratory Diagnostic of the “Dr. Juraj Njavro” National Memorial Hospital Vukovar. Ethical approval for the collection and processing of the biological samples and the acquisition of medical documentation was obtained from the Ethics Committees of University Hospital Osijek (number: R1-8243/2023) and National Memorial Hospital “Dr. Juraj Njavro” Vukovar (cl: 510-05/23, registration number: 107-16-23-08-04). The privacy and confidentiality of all participant data were rigorously maintained throughout the study. The blank English-language informed consent form and raw data are available upon request and have been submitted alongside the manuscript. The raw data are available as supplementary materials as well.

### 2.1. Participants

All participants included in the study were of southeastern European descent. This study included 162 pregnant women in the third trimester of pregnancy (27–40 weeks of gestation), categorized into four distinct groups. The healthy controls (Group 1) comprised women with normal glucose and HbA1c values, negative OGTT results in the current pregnancy, and no history of GD, other pregnancy pathologies, pre-existing diabetes, or known autoimmune or metabolic diseases. The healthy controls with comorbidities (Group 2) included women with normal glucose, HbA1c, and OGTT results in the current pregnancy, but who had a history of GD in a previous pregnancy, and/or pre-existing autoimmune diseases, blood pressure disorders, polycystic ovary syndrome (PCOS), or obesity, or had developed any of these comorbidities during the current pregnancy. Women diagnosed with GD during the current pregnancy according to the IADP-SG guidelines, with no other pregnancy complications, formed the GD group (Group 3). Finally, the GD with complications group (Group 4) consisted of women diagnosed with GD in the current pregnancy (IADP-SG criteria) who also presented with preeclampsia, high blood pressure, and/or a pre-existing or current immune system disease. Participants were excluded if they did not provide informed consent or had a pre-existing diagnosis of diabetes or other known metabolic diseases outside of pregnancy.

### 2.2. Data Collection, Blood Sampling, and Laboratory Analysis

Sociodemographic and clinical data were collected in cooperation with the pregnant women, and available medical documentation about their pregnancy was also used. This included information on pre- and during-pregnancy physical status, family and personal medical history, dietary habits, lifestyle factors, and prior pregnancy events.

A single venous blood sample was collected from each participant in the third trimester of pregnancy, following the acquisition of informed consent. Blood samples were collected from participants in a fasting state between 7:00 and 8:00 a.m. Peripheral venous blood was drawn into one K3EDTA tube and one serum tube using BD Vacutainers (Beckton Dickinson, Franklin Lakes, NJ, USA).

The profiles of lymphocyte subsets, including B-lymphocytes (CD19+), helper T-lymphocytes (CD3+CD4+), cytotoxic T-lymphocytes (CD3+CD8+), natural killer (NK) cells (CD3-CD16+56+), and natural killer T (NKT) cells (CD3+CD16+56+), were determined via flow cytometry using a BD FACSLyric analyzer (Beckton Dickinson, Heidelberg, Germany) on whole EDTA blood. The cell surface expression of specific clusters of differentiation (CD) markers (antigens) was analyzed using commercial fluorochrome-conjugated monoclonal antibodies: BD Multitest CD3 FITC/CD8 PE/CD45 PerCP/CD4 APC and BD Multitest CD3 FITC/CD16+56 PE/CD45 PerCP/CD19 APC (Beckton Dickinson, Heidelberg, Germany).

The biochemical and immunochemical parameters were measured in serum samples using a cobas pro analyzer (Roche Diagnostics, Rotkreuz, Switzerland). We measured the glucose, insulin, C-peptide, hemoglobin A1c, C-reactive protein, immunoglobulin G, immunoglobulin M, immunoglobulin A, iron, ferritin, vitamin B12, folic acid, cholesterol, HDL cholesterol, LDL cholesterol, triglycerides, and NT-proBNP.

### 2.3. Flow Cytometry

Samples were prepared for analysis according to the reagent manufacturer’s instructions. A total of 100 µL of whole EDTA blood of the subject and 20 µL of antibodies were added to a clean test tube and vortexed. The mixture was incubated for 20 minutes at room temperature in the dark, and then 2 mL of BD Lyse Solution was added and the incubation in the dark at room temperature was repeated for 10 minutes. The mixture was then centrifuged for 5 minutes at 300× *g* and the supernatant was separated. Following this, 2 mL of BD Wash solution was added to the sediment, and centrifugation was repeated for 5 minutes at 300× *g*. After centrifugation, the supernatant was separated and 250 µL of BD Wash solution was added to the sediment and vortexed, and the samples were placed on the BD FACSLyric analyzer (Beckton Dickinson, Heidelberg, Germany) with the anticoagulant K3EDTA.

A detailed gating strategy was employed to identify specific lymphocyte subsets. Initially, lymphocytes were identified based on their characteristic forward (FSC) and side scatter (SSC) properties. Doublets were excluded using FSC-A vs. FSC-H plots, and dead cells were subsequently excluded using a live/dead discriminator dye. Within the live lymphocyte gate, T lymphocytes were defined as CD3+. From this population, helper T lymphocytes were identified as CD3+CD4+, and cytotoxic T lymphocytes as CD3+CD8+. NK cells were characterized as CD3−CD16+56+, while NKT cells were defined as CD3+CD16+56+. For B lymphocytes, the CD19+ marker was used, with B1 lymphocytes specifically identified as CD19+CD5+, and B2 lymphocytes as CD19+CD5−. Ratios were then calculated based on the percentages of these identified cell populations.

### 2.4. Statistical Analysis

The statistical packages Med Calc Statistical Software version 14.12.0 (MedCalc Software bvba, Ostend, Belgium; http://www.medcalc.org; 2014) and SPSS Statistics 23 (IBM Corp. Released 2015. IBM SPSS Statistics) were used for statistical analysis.

To detect a medium effect (f = 0.25) in the difference in numerical variables between four independent groups of respondents, with a significance level of 0.05 and a power of 0.80, the minimum required sample size is 159 respondents. Calculations were made using the program G*Power version 3.1.2, Franz Faul, University of Kiel, Germany, before the start of the research.

The categorical data are represented by the absolute and relative frequencies. Numerical data are described by the median and the limits of the interquartile range. Differences in categorical variables were tested with Fisher’s exact test. The normality of the distribution of numerical variables was tested with the Shapiro–Wilk test. Differences in numerical variables between two independent groups were tested with the Mann–Whitney U-test. Differences in normally distributed numerical variables for more than two independent groups were tested using the Kruskal–Wallis test (post hoc Conover). The association between numerical variables was assessed using Spearman’s correlation coefficient (Rho). All *p* values were two-sided. The significance level was set at 0.05.

## 3. Results

### 3.1. Age and Pre-Pregnancy BMI Differences Among Study Groups

The study cohort comprised 162 participants, categorized into four distinct groups; 40 (24.7%) of the participants were in the groups without GDM and other complications, 40 were in the group without GDM but with complications that may affect the immune profile, 40 were in the group of subjects with GD and complications that may affect the immune profile, and 42 (25.9%) were in the group with only GD. The median age of the entire study population was 31 years, with an age range spanning from 19 to 48 years. Statistical analysis revealed that participants with GD were significantly older compared to the group of pregnant women without GD (Mann–Whitney U-test, *p* = 0.005). Furthermore, a pre-pregnancy body mass index (BMI) exceeding 30 kg/m^2^ was significantly more prevalent in the group with GD (Fisher’s exact test, *p* < 0.001) (Table 1).

### 3.2. Variations in the Analyzed Ratios Between Women with GD and Healthy Pregnant Women

Analysis of the ratios of NKT/NK cells, B1/B2 lymphocytes, and helper/cytotoxic lymphocytes revealed no statistically significant differences between pregnant women with gestational diabetes (GD) and healthy pregnant women (Table 2). This lack of significant variation persisted regardless of the presence of immunological complications during pregnancy within either group.

### 3.3. Correlations Between Biochemical Indicators and Immune Cell Ratios in the Entire Cohort

The analysis of the correlations between biochemical indicators and immune cell ratios across the entire study cohort revealed several significant associations. The NKT/NK cell ratio demonstrated a positive and statistically significant correlation with the concentrations of C-peptide (Spearman’s Rho = 0.226, *p* < 0.05) and triglycerides (Rho = 0.207, *p* < 0.05). Conversely, a negative and statistically significant correlation was observed between the NKT/NK cell ratio and the concentration of high-density lipoprotein cholesterol (HDL cholesterol) (Rho = −0.232, *p* < 0.05). The ratio of helper to cytotoxic lymphocytes was found to be negatively and significantly correlated with the concentrations of insulin (Rho = −0.235, *p* < 0.05) and C-peptide (Rho = −0.190, *p* < 0.05) (Table 3).

### 3.4. Correlations Between Biochemical Indicators and Immune Cell Ratios in Pregnant Women with GD

The analysis of the correlations between biochemical indicators and immune cell ratios revealed specific significant associations within the subgroup of participants diagnosed with GD. The NKT/NK cell ratio exhibited a positive and statistically significant correlation with the concentration of C-peptide (Rho = 0.232, *p* < 0.05). The helper/cytotoxic lymphocyte ratio showed a negative and statistically significant correlation with the concentration of insulin (Rho = −0.276, *p* < 0.05), while it demonstrated a positive and statistically significant correlation with the concentration of folic acid (Rho = 0.234, *p* < 0.05) (Table 4).

### 3.5. Correlations Between Biochemical Indicators and Immune Cell Ratios Between Groups

The correlations between the ratios of NKT/NK cells, B1/B2 lymphocytes, and helper/cytotoxic lymphocytes with biochemical and immunochemical parameters that proved to be significant in each group are shown in Table 5.

## 4. Discussion

The aim of this cross-sectional study was to investigate whether there were differences in the ratios of NKT/NK cells, B1/B2 lymphocytes, and helper/cytotoxic lymphocytes between pregnant women with GD and healthy pregnant women. The results were compared between pregnant women who did not develop GD and pregnant women with GD and in smaller groups among four groups of pregnant women: healthy pregnant women (not diagnosed with diabetes) with and without complications, and pregnant women with GD and with and without other complications. In recent years, there has been an increasing amount of research on this topic, but a review conducted several years ago showed that there was no research including the profile of B-lymphocytes. It is assumed that pregnant women’s immune system and its dysfunction could play a major role in the development of insulin resistance and damage to pancreatic β-cells.

The collected general data on pregnant women show that in our study, pregnant women with GD are older than pregnant women who were not diagnosed with GD during pregnancy, which is in line with the expectations and current knowledge on GD. A large meta-analysis published in 2022 that included over 120 million respondents showed a strong association between the risk of developing GD and maternal age, with a linear increase, and proposed a cut-off age of 25 years for GD screening in pregnant women [20]. Laine et al. demonstrated in their cohort study that primiparous women older than 35 years have a three-fold higher risk of developing GD than primiparous women younger than 25 years [21]. BMI is considered a risk factor for the development of GD, and this is represented in almost every study. What was also shown in this study is that pregnant women with GD have a higher BMI than healthy pregnant women. There was a statistically significant difference in pregnant women with GD and complications compared to the remaining groups. Mirabelli et al. demonstrated in their study that maternal BMI before pregnancy plays a key role in the development of GD. BMI is an indicator of visceral obesity and systemic insulin resistance and is a modifying risk factor for GD; therefore, Mirabelli et al. suggest it as a suitable target for raising public awareness of GD [22]. In the group of women with GD and complications, a family history of diabetes, endocrine diseases and high blood pressure before pregnancy or during a previous pregnancy, and problems with miscarriage are also more common. It has been shown in previous studies that women with hypertension, either during the five years before pregnancy or during the first trimester of pregnancy, have a two-fold higher risk of developing GD during pregnancy, and this association is independent of the woman’s BMI. However, the association between blood pressure and GD was stronger among overweight women [23].

Our study shows that there is no statistically significant difference in the ratios of NKT/NK cells, B1/B2 lymphocytes, and helper/cytotoxic lymphocytes between pregnant women with GD and healthy pregnant women, regardless of immunological complications during pregnancy.

When linking biochemical indicators and ratios, it was observed in all subjects that the NKT/NK cell ratio was positively and significantly correlated with the concentrations of C-peptide (*p* < 0.01) and triglycerides (*p* = 0.01), while there was a negative relationship with the concentration of HDL cholesterol (*p* < 0.01). The positive correlation of NKT/NK cells with the concentration of C-peptide and triglycerides indicates the connection of metabolic processes with the immune system. Elevated values of C-peptide and triglycerides may be associated with insulin resistance, which is physiologically increased in pregnancy, and the positive relationship of the NKT/NK cell ratio could indicate a connection of the immune system with insulin resistance. Given the protective role of HDL cholesterol and the fact that it is reduced in inflammatory states, its negative correlation with the NKT/NK cell ratio is expected. Certain NKT cell subsets can influence insulin sensitivity and lipid metabolism through the secretion of both pro-inflammatory (and anti-inflammatory) cytokines. These cytokines can directly impact adipocyte function, hepatic glucose production, and pancreatic β-cell insulin secretion. For instance, an altered NKT cell activation state or cytokine profile could contribute to the chronic low-grade inflammation often associated with insulin resistance in metabolic disorders, including GD. Also, NKT cells are unique in their ability to respond to lipid antigens, which can be presented by CD1d molecules on various cell types, including adipocytes. Their activation and subsequent cytokine release can be modulated by dietary lipids and ligands originating from the gut microbiome, further emphasizing their role at the nexus of immunity and metabolism [24]. This aligns with the observed associations, suggesting that NKT cells may be actively involved in sensing and responding to alterations in lipid metabolism, which in turn influences systemic inflammation and insulin resistance. All of this is in accordance with this positive association with C-peptide and negative association with HDL cholesterol. Our observed correlation between the NKT/NK ratio and metabolic markers like C-peptide/triglycerides is indeed intriguing and points to a potential direct interplay between immune cells and metabolic regulation in GD.

In the group of pregnant women with GD and complications, the NKT/NK cell ratio was also positively correlated with the insulin concentration (*p* = 0.01). NKT cells can affect insulin production and gluconeogenesis in the liver through the production of various cytokines and the function of adipose tissue, which includes fat storage and adipokine production, but they can also modulate the activity of other immune cells involved in the regulation of glucose metabolism. However, increased NKT cell activity can also increase inflammatory processes and thereby increase insulin resistance, causing the pancreas to produce larger amounts of insulin. In our study, a reduced percentage of NK cells was found in pregnant women with diabetes compared to healthy pregnant women, which is consistent with the results obtained [25]. This association is also consistent with the positive association between the NKT/NK cell ratio and C-peptide among all groups studied (*p* < 0.05), given the equivalent role of C-peptide and insulin.

The helper/cytotoxic lymphocyte ratio is negatively and significantly correlated with insulin (*p* < 0.01) and C-peptide (*p* = 0.02) concentrations. In pregnant women with GD, regardless of complications, this ratio was positively correlated with folic acid (*p* = 0.03). The existing literature extensively demonstrates that folate influences DNA methylation patterns in immune cells, thereby modulating gene expression profiles related to immune cell function and differentiation. For example, studies have shown that folate supplementation can modulate the Th1/Th2 balance in certain inflammatory conditions by influencing the methylation status of cytokine gene promoters. This mechanistic understanding from the broader immunology field provides strong biological plausibility for our observed association between folic acid levels and the helper/cytotoxic T cell ratio in GD patients [26]. Growing interest in the relationship between folate and B12 status and GD has revealed that high maternal folate levels combined with low B12 concentrations are associated with an increased risk of GD and insulin resistance in the offspring [27]. Although the precise mechanisms remain to be explored, hypotheses include a methyl trap leading to elevated homocysteine levels, disrupted methylation reactions, and alterations in mitochondrial metabolism. Furthermore, supplementing pregnant women with high doses of folic acid can lead to the appearance of unmetabolized folic acid (UMFA) in plasma [27]. The presence of UMFA is associated with altered NK cell cytoactivity, an immune dysregulation implicated in GD pathology through altered cell infiltration and signaling pathways. The characteristic insulin resistance and low-grade chronic inflammation prevalent in gestational diabetes may alter folate bioavailability or its enzymatic pathways, affecting its ability to maintain immune cell homeostasis and T cell differentiation. Specifically, an optimized folate status could support balanced T cell subsets, which is critical considering the proven link between immune dysregulation and metabolic dysfunction in GD. Thus, the correlation we observed between folic acid and the helper/cytotoxic T cell ratio provides insight into how nutritional factors may modulate T cell profiles in GD, directly linking to the broader metabolic disturbances characteristic of GD and potentially contributing to its pathophysiology.

Helper and cytotoxic T-lymphocytes have different roles in the immune response; helper T-lymphocytes coordinate the immune response, while cytotoxic T-lymphocytes destroy inflammatory cells and NKT cells have a regulatory role [28]. The negative correlation suggests that there is a certain balance between these populations of immune cells. Increasing the percentage of NKT cells may lead to a decrease in the helper/cytotoxic lymphocyte ratio, which may indicate a redirection of the immune response; that is, it is possible that NKT cells inhibit the activation and proliferation of T-lymphocytes, thus leading to a reduced percentage of helper lymphocytes. An increased number of cytotoxic T-lymphocytes may be associated with an increased inflammatory response, leading to tissue damage and the development of insulin resistance, while a decrease in the number of helper T-lymphocytes may lead to the weakening of anti-inflammatory protection. All these factors contribute to the development of insulin resistance and increased concentrations of insulin and C-peptide.

The B1/B2-lymphocyte ratio did not show significant correlations with biochemical parameters; the only negative correlation was found between the B1/B2-lymphocyte ratio and ferritin in the group of healthy pregnant women with complications (*p* = 0.03). A decrease in the B1/B2-lymphocyte ratio, or an increase in the proportion of B2 lymphocytes, may be associated with increased iron requirements, which could lead to a decrease in ferritin. It has been proven that iron deficiency has a clear effect on lymphocyte subsets. Changes in lymphocyte subsets mainly begin in response to decreased hemoglobin concentrations, rather than to iron or ferritin deficiency. The synchronized decline in hemoglobin and increase in the total iron-binding capacity led to an absolute decrease in total lymphocytes, mainly NK cells, and a relative increase in T-lymphocytes, mainly helper T-lymphocytes [29]. On the other hand, complications in pregnancy are often associated with inflammatory processes that can affect iron metabolism and lead to a decrease in ferritin levels.

## 5. Conclusions

There was no statistically significant difference in the ratios of NKT/NK cells, B1/B2 lymphocytes, and helper/cytotoxic lymphocytes between pregnant women with GD and healthy pregnant women, regardless of immunological complications during pregnancy. In all participants, the ratio of NKT/NK cells was positively and significantly related to the concentrations of C-peptide and triglycerides, while the relationship with the concentration of HDL cholesterol was negative. In pregnant women with GD and complications, the NKT/NK cell ratio was positively correlated with insulin concentration. The helper/cytotoxic lymphocyte ratio was negatively and significantly correlated with the concentrations of insulin and C-peptide. In pregnant women with GD, regardless of complications, this ratio was positively correlated with the measured values of folic acid. The B1/B2-lymphocyte ratio did not show significant correlations with biochemical parameters, where the only negative correlation was found between the B1/B2-lymphocyte ratio and ferritin in the group of healthy pregnant women with complications.

Despite the use of standardized methods such as flow cytometry, our experimental design has certain limitations. A significant concern is the inability to fully adjust for all potential confounding variables such as age, pre-pregnancy body mass index, and maternal health status. These factors are well-established modulators of immune function and metabolic health, both of which are critical for the pathogenesis of GD. Future studies should aim for larger, multicenter cohorts to allow for robust statistical adjustment for a comprehensive set of demographic and clinical covariates, thereby increasing the generalizability and reliability of the findings.

Furthermore, a key limitation of this work is its cross-sectional design, which inherently precludes the establishment of causal inferences. The observed correlations might reflect reverse causality or be influenced by shared underlying factors not fully captured in our analysis. To address this, future research should incorporate longitudinal study designs to track immune-metabolic changes over time, particularly during critical periods like postpartum. Ultimately, interventional studies will be crucial to definitively determine the causal impact of specific immune or metabolic parameters on GD progression and to inform potential therapeutic strategies.

We carefully stratified groups with pre-existing conditions and major obstetric complications to isolate the effects of GD to ensure that the observed immunological differences were primarily attributable to GD itself and not to concomitant conditions.

## Figures and Tables

**Table 1:** General characteristics of participants.

	Number (Percentage) of Participants					
	Group 1	Group 2	Group 3	Group 4	Summary	*p*
Age (years)	29	31	33	31	31	0.01 ^†§^
[median (IQR)]	(28–32)	(27–35)	(29–37)	(29–36)	(28–35)	
Pre-pregnancy BMI						<0.001 ^‡^
<19	3 (7.5)	3 (7.5)	1 (2.4)	0 (0)	7 (4.3)	
19–24.9	36 (90)	20 (50)	18 (42.9)	10 (25)	84 (51.9)	
25–29.9	1 (2.5)	14 (35)	11 (26.2)	15 (37.5)	41 (25.3)	
>30	0 (0)	3 (7.5)	12 (28.5)	15 (37.5)	30 (18.5)	

IQR—interquartile range; BMI—body mass index; ^†^ Kruskal–Wallis test (post hoc Conover), ^§^ *p* < 0.05 for (1) vs. (3, 4); and ^‡^ Fisher test.

**Table 2:** Differences in NKT/NK cell, B1/B2 lymphocyte, and helper/cytotoxic lymphocyte ratios between the studied groups.

	Median (IQR)				
	Group 1	Group 2	Group 3	Group 4	*p* *
NKT/NK cells	0.461	0.689	0.72	0.65	0.10
	(0.24–0.84)	(0.37–1.51)	(0.38–1.45)	(0.39–1.26)	
B1/B2 lymphocytes	0.26	0.23	0.21	0.29	0.25
	(0.14–0.4)	(0.15–0.4)	(0.12–0.3)	(0.16–0.5)	
Helper/cytotoxic lymphocytes	1.65	1.48	1.46	1.78	0.10
	(1.32–2)	(1.22–2)	(1.23–1.9)	(1.46–2.2)	

IQR—interquartile rangeand * Kruskal–Wallis test (post hoc Conover).

**Table 3:** The correlation of NKT/NK cells, B1/B2 lymphocytes, and helper/cytotoxic lymphocytes with immunochemical and biochemical parameters in all participants.

	Spearman’s Rho (*p* Value)		
	NKT/NK Cells	Helper/Cytotoxic Lymphocytes	B1/B2 Lymphocytes
Glucose	0.018 (0.82)	−0.005 (0.95)	0.034 (0.67)
Insulin	0.145 (0.07)	**−0.235 (<0.001)**	0.080 (0.31)
C-peptide	**0.226 (<0.001)**	**−0.190 (0.02)**	0.001 (0.99)
Hemoglobin A1c	0.036 (0.65)	0.017 (0.83)	−0.006 (0.94)
C-reactive protein	0.140 (0.08)	−0.081 (0.31)	0.027 (0.74)
Immunoglobulin G	0.051 (0.52)	−0.104 (0.19)	−0.003 (0.97)
Immunoglobulin M	−0.084 (0.29)	−0.031 (0.70)	0.001 (0.99)
Immunoglobulin A	0.023 (0.77)	−0.010 (0.90)	−0.019 (0.81)
Iron	−0.104 (0.19)	−0.075 (0.34)	−0.107 (0.18)
Ferritin	0.061 (0.44)	0.049 (0.54)	−0.097 (0.22)
Vitamin B12	−0.119 (0.13)	−0.047 (0.56)	0.021 (0.79)
Folic acid	−0.149 (0.06)	0.105 (0.18)	0.020 (0.80)
Cholesterol	0.013 (0.87)	−0.009 (0.91)	0.016 (0.84)
HDL cholesterol	**−0.232 (<0.001)**	0.102 (0.20)	0.036 (0.65)
LDL cholesterol	0.018 (0.82)	−0.044 (0.58)	−0.020 (0.80)
Triglycerides	**0.207 (0.01)**	−0.046 (0.56)	−0.032 (0.69)
NT-proBNP	−0.112 (0.16)	0.139 (0.08)	−0.054 (0.50)

Bolded values indicate statistically significant differences.

**Table 4:** The correlation of NKT/NK cells, B1/B2 lymphocytes, and helper/cytotoxic lymphocytes with immunochemical and biochemical parameters in pregnant women with GD.

	Spearman’s Rho (*p* Value)		
	NKT/NK Cells	Helper/Cytotoxic Lymphocytes	B1/B2 Lymphocytes
Glucose	0.099 (0.38)	−0.078 (0.49)	0.016 (0.89)
Insulin	0.208 (0.06)	**−0.276 (0.01)**	0.164 (0.14)
C-peptide	**0.232 (0.04)**	−0.208 (0.06)	0.040 (0.72)
Hemoglobin A1c	0.113 (0.32)	−0.049 (0.66)	−0.035 (0.76)
C-reactive protein	−0.032 (0.78)	0.008 (0.94)	0.044 (0.70)
Immunoglobulin G	0.06 (0.59)	−0.182 (0.10)	−0.032 (0.77)
Immunoglobulin M	0.011 (0.92)	−0.170 (0.13)	−0.039 (0.73)
Immunoglobulin A	0.055 (0.62)	−0.013 (0.91)	−0.014 (0.90)
Iron	−0.200 (0.07)	0.018 (0.87)	−0.095 (0.40)
Ferritin	−0.087 (0.44)	0.137 (0.22)	−0.061 (0.59)
Vitamin B12	−0.183 (0.10)	−0.04 0(0.72)	0.165 (0.14)
Folic acid	−0.208 (0.06)	**0.234 (0.03)**	0.115 (0.30)
Cholesterol	0.049 (0.66)	0.011 (0.92)	−0.073 (0.51)
HDL cholesterol	−0.171 (0.13)	0.095 (0.40)	−0.020 (0.86)
LDL cholesterol	0.085 (0.45)	−0.070 (0.53)	−0.092 (0.41)
Triglycerides	0.128 (0.25)	0.079 (0.48)	−0.016 (0.88)
NT-proBNP	−0.128 (0.25)	0.101 (0.37)	−0.145 (0.19)

Bolded values indicate statistically significant differences.

**Table 5:** The correlation of NKT/NK cells, B1/B2 lymphocytes, and helper/cytotoxic lymphocytes with immunochemical and biochemical parameters in pregnant women between groups.

Test Group	Parameter	Spearman’s Rho (*p* Value)		
		NKT/NK Cells	Helper/Cytotoxic Lymphocytes	B1/B2 Lymphocytes
Group 1	Iron	0.061 (0.71)	**−0.323 (0.04)**	−0.108 (0.51)
Group 2	C-reactive protein	0.262 (0.10)	**−0.383 (0.01)**	−0.076 (0.64)
	Ferritin	0.092 (0.57)	−0.145 (0.37)	**−0.350 (0.03)**
	HDL cholesterol	**−0.468 (<0.001)**	0.241 (0.13)	−0.028 (0.87)
	Triglycerides	0.243 (0.13)	**−0.329 (0.04)**	−0.032 (0.84)
Group 3	Insulin	0.073 (0.65)	**−0.353 (0.02)**	0.030 (0.85)
	C-peptide	0.256 (0.10)	**−0.340 (0.03)**	−0.140 (0.38)
Group 4	Insulin	**0.412 (0.01)**	−0.317 (0.05)	0.273 (0.09)
	Immunoglobulin G	0.023 (0.89)	**−0.450 (<0.001)**	0.062 (0.71)
	Immunoglobulin A	0.243 (0.14)	**−0.338 (0.03)**	−0.095 (0.56)
	Iron	**−0.355 (0.03)**	0.183 (0.26)	−0.085 (0.60)
	Folic acid	**−0.345 (0.03)**	**0.374 (0.02)**	−0.056 (0.73)

Bolded values indicate statistically significant differences.

## Data Availability

The data that support the findings of this study are available in the Supplementary Materials.

## Supplementary Materials

The following supporting information can be downloaded at: https://www.mdpi.com/article/10.3390/metabo15060378/s1, Table S1: GD project raw data.
